# Development of a 3D finite element model of lens microcirculation

**DOI:** 10.1186/1475-925X-11-69

**Published:** 2012-09-19

**Authors:** Ehsan Vaghefi, Duane TK Malcolm, Marc D Jacobs, Paul J Donaldson

**Affiliations:** 1 Department of Optometry and Vision Sciences, University of Auckland, Building 502, Level 4, 85 Park Road, Grafton, Auckland, New Zealand; 2 Auckland Bioengineering Institute, University of Auckland, Auckland, New Zealand; 3 School of Medical Sciences, University of Auckland, Auckland, New Zealand

**Keywords:** Computational modelling, Ocular lens, Microcirculation, Finite element, Physiological optics

## Abstract

**Background:**

It has been proposed that in the absence of a blood supply, the ocular lens operates an internal microcirculation system. This system delivers nutrients, removes waste products and maintains ionic homeostasis in the lens. The microcirculation is generated by spatial differences in membrane transport properties; and previously has been modelled by an equivalent electrical circuit and solved analytically. While effective, this approach did not fully account for all the anatomical and functional complexities of the lens. To encapsulate these complexities we have created a 3D finite element computer model of the lens.

**Methods:**

Initially, we created an anatomically-correct representative mesh of the lens. We then implemented the Stokes and advective Nernst-Plank equations, in order to model the water and ion fluxes respectively. Next we complemented the model with experimentally-measured surface ionic concentrations as boundary conditions and solved it.

**Results:**

Our model calculated the standing ionic concentrations and electrical potential gradients in the lens. Furthermore, it generated vector maps of intra- and extracellular space ion and water fluxes that are proposed to circulate throughout the lens. These fields have only been measured on the surface of the lens and our calculations are the first 3D representation of their direction and magnitude in the lens.

**Conclusion:**

Values for steady state standing fields for concentration and electrical potential plus ionic and fluid fluxes calculated by our model exhibited broad agreement with observed experimental values. Our model of lens function represents a platform to integrate new experimental data as they emerge and assist us to understand how the integrated structure and function of the lens contributes to the maintenance of its transparency.

## Background

**Figure 1:**
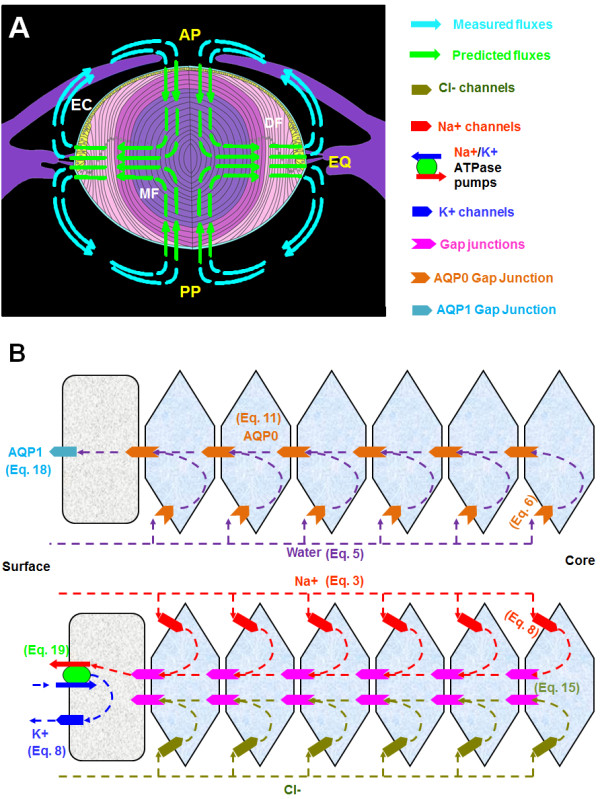
**Lens microcirculation system.** A: Schematic diagram of an axial view of the lens showing a single layer of anterior epithelial monolayer (EC), that at the equator (EQ) divide to produce the elongating differentiating fibre cells (DF), that eventually lose their nuclei and cellular organelles to become mature fibre cells in the lens nucleus (MF). Fiber cells from adjacent hemispheres meet at the anterior (AP) and posterior (PP) poles to form the sutures. Arrows in the diagram represent the direction of ion and water fluxes. These fluxes have been directly measured outside the lens [5,6] but their position and direction inside the lens are to date purely theoretical [3]. B: An equatorial cross section of the lens showing a cellular view of ion and water movement in the lens [7]. Current and solutes are proposed to flow into the lens via the extracellular space, to cross fibre cell membranes, and to flow outward via an intracellular pathway mediated by gap junction channels.

Our sense of sight is critically dependant on the ability of the optical pathway, formed by the cornea and lens, to focus light onto the retina. As an optical element in this pathway, the lens needs to maintain its transparency and create sufficient optical power. To generate its required optical properties, the lens has evolved a unique structure to minimise light scattering and enhance transparency [1] [see Figure 1. However, the lens is not a purely passive optical element and its structure and therefore optical properties need to be actively maintained. It has been proposed by Mathias et al. [2] that in the absence of a blood supply, the lens operates an internal microcirculation system. This system delivers nutrients, removes wastes and maintains the ionic homeostasis of the lens [3,4]. Hence, this system is critical for maintaining the optical properties of the lens.

The microcirculation model was initially based on a combination of electrical impedance measurements and theoretical modelling [8-11]. The observation that the measured ionic currents were directed inward at the poles and outward around the equator [Figure 1A, led to the suggestion that these currents represent the external portion of a circulating ionic current that drives the internal microcirculatory system within the lens. Briefly, the working model is that the current, carried primarily by Na^+^, enters at all locations around the lens via the extracellular space between fibre cells. Na^+^ eventually crosses the fibre cell membranes, and then flows from cell-to-cell towards the surface via an intracellular pathway mediated by gap junction channels [Figure 1B. The gap junction coupling conductance in the outer shell of differentiating fibres is concentrated at the equator [12,13]. Hence, the intracellular current is directed to the equatorial epithelial cells. Here, the highest densities of Na^+^/K^+^ pumps are located to actively transport Na^+^ out of the lens [14]. Thus, the intracellular current effluxes are highly concentrated at the equator causing the net current to be outward. At the poles, there is very little intracellular current so the net current is predominantly inward, along the extracellular spaces [Figure 1B. The driving force for these fluxes is hypothesized to be the difference in the electromotive potential of surface cells that contain Na^+^/K^+^ pumps and K^+^-channels, and inner fiber cells that lack functional Na^+^/K^+^ pumps and K^+^-channels and whose permeability is dominated by non-selective cation and Cl^-^ conductances [15]. This electrical connection together with the different membrane properties of the surface and inner cells causes the standing current to flow. In this model, the circulating current creates a net flux of solute that in turn generates fluid flow. The extracellular flow of water convects nutrients towards the deeper lying fiber cells, while the intracellular flow removes wastes and creates a well-stirred intracellular compartment.

Thus in this model, it is the circulating Na^+^ current that generates a circulation of fluid inside the lens. It is important to note that while the existence of the circulating ionic currents are firmly based on existing experimental data, circulating fluid flows in the lens have proven more difficult to measure. At present, these fluxes are only predicted to occur from indirect measurements and models of the measured electrical properties of the lens. The current model of the lens fluid dynamics is based on an equivalent circuit analysis of the microcirculation system [3,16]. This analytical model inherently relied on approximate solutions and simplification of the underlying physics. To improve our understanding of the circulation system, here we have adopted a finite element modelling (FEM) approach. Using FEM, we have developed a 3D computer model of the microcirculation system that encapsulates the complex interplay of its important features and can also be solved numerically. This computer-based modelling approach allowed structural features such as fiber cell orientation, extracellular space dimensions and gap junction distribution to be included in the model. It also included functional information on the spatial differences in membrane permeability between surface and inner cells, thought to drive the circulating currents.

In this paper we describe our expansion of the equations that govern ion and fluid dynamics in the lens [2,3,10,15-18] to 3D, and their subsequent implementation into a new FEM that encapsulates known structural and functional parameters of the mouse lens. This model is based on our continuous imaging and modelling iterative investigation into the fluid dynamics of the lens [19-25]. To test the ability of this computer model to reproduce the functional properties of the lens, we have used a series of experimentally derived boundary conditions [26-28] to allow it to be solved. We show that our model is capable of predicting the experimentally measured steady state lens properties and to generate circulating ion and water fluxes. Hence we believe that our computer model is a useful tool to study how lens structure and function influence its optical properties.

## Methods

In this section, we first present the derivation of the fundamental mathematical equations, originally formulated by Mathias et al. to describe the microcirculation system [2,3,10,15-18]. We then develop a computer mesh to represent the structure of the mouse lens to enable these equations to be implemented in 3D. Next we implement the model using the C++ programming language and solve the model using experimentally derived boundary conditions [26-28]. The resultant 3D model calculates ion concentration gradients, membrane potential gradients, and intra- and extracellular ion and water fluxes in different regions of the lens.

### Derivations of general equations

The microcirculation model has been based on a distinct yet mutually dependent movement of water and ions [3,4,15,16,29]. Simplified derivations of the Navier–Stokes equations and the advective Nernst-Plank equations [30,31] where used to represent water and ion fluxes, respectively. All symbols used in these and the resultant equations are listed in [Table 1.

**Table 1:** Glossary of symbols used in this manuscript

**Symbol**	**Description**	**Units**
C	concentration	mol/ m^3^
D	diffusion coefficient	m^2^/s
R	gas constant	J/ (mol.K)
e	electron charge	C
E	Nernst potential	V
F	Faraday constant	C/mol
h	extracellular cleft width	m
I_max_	maximum Na/K ATPase pump current density	A/cm^2^
I_p_	Na/K ATPase pump current density	A/cm^2^
j_m_	transmembrane flux	mol/ (m^2^s)
g	conductivity per membrane area	S/m^2^
k_B_	Boltzmann constant	J/K
K_0.5_	half-maximal concentrations	mol/ m^3^
L_s_	surface hydraulic permeability	m^3^/(N.s)
L_p_	intercellular hydraulic permeability	m^3^/(N.s)
Os	osmolarity	Osm/L
p	pressure	Pas
P_m_	membrane solute permeability	m/s
r	equatorial radius of the model	m
a	posterior radius of the model	m
b	anterior radius of the model	m
s_α_	solute source	mol/(m^3^s)
s	fluid mass source	1/m
T	temperature	K
V_m_	transmembrane potential	V
f	body force	N/Kg
z	valence	-
α	solute species	-
ε_0_	permittivity of vacuum	F/m
ε_r_	dielectric constant	S/m
ζ	zeta potential (cell membrane potential)	V
λ_D_	Debye length	m
Λ	volume fraction	-
μ	dynamic viscosity	N.s/m^2^
ρ	mass density	Kg/m^3^
ρ_m_	membrane density	1/m
σ	membrane reflectance	-
ϕ	potential	V
u	velocity	m/s

#### Fluid fluxes

The Navier–Stokes equations are derived from the conservation of mass, momentum, and energy. The nonlinearity of these equations made them challenging to be solved directly. However, this set of equations can be simplified with the right assumptions for a given system. We assumed the lens’s water to be an incompressible Newtonian fluid with a spatially constant viscosity at steady state. Also, we assumed that the lens fluid flow can be described as a “creeping” low-Reynolds number flow with ignorable turbulence [32-34]. Considering these assumptions, we used the simplified form of the general Navier–Stokes equations, called the Stokes equations [Eq. 1, Eq. 2.

(1) ∇.u=0

(2) $$-\nabla p+\mu \nabla^{2}u+\text{pf}=0$$

The parameters and their units are listed in [Table 1. We used these equations to model the movement of water in the lens. All the fluid flow related constants utilized in this model are listed in [Table 2. Most of the parameters are similar to the parameters used in the analytic model [2,3,10,15-18]. The most significant change between the analytic and numerical models is the intercellular, surface and fibre cell hydraulic permeabilities, where more recent values, which were measured by [35], are used.

**Table 2:** Fluid flow parameters

**Symbol**	**Description**	**Value**	**Units**
R	Gas-constant	8.314 × 10^3^	pJ/(nmol.K)
ε_0_	Permittivity-of-vacuum^5^	8.854	pF/m
ε_r_	Dielectric-constant-(water)^5^	80.4	
$\tilde{h}$	Extracellular-cleft-width^2^	40	nm
μ	Fluid-viscosity	700	mPa.ms
ζ	Zeta-potential^1^	−15	mV
λ_D_	Debye-length^2^	1	nm
L_p_	Intercellular-hydraulic-permeability^3^	4.0 × 10^−8^	m/(Pa.s)
L_f_	Fibre-cell-membrane-hydraulic-permeability^3^	4.0 × 10^−8^	m/(Pa.s)
L_s_	Surface-hydraulic-permeability^3^	2.7 × 10^−7^	m/(Pa.s)
σ	Intercellular-membrane-reflectance^1^	1	
$\tilde{K}$_e_	Extracellular-hydraulic-conductivity	3.05 × 10^−14^	m^2^/(Pa.s)
$\tilde{k}$_e_	Extracellular-electro-osmotic-coefficient	1.45 × 10^−8^	m^2^/(Vs)

References: 1. Mathias (1985) [2]; 2. McLaughlin & Mathias (1985) [36]; 3. Varadaraj et al. (2005) [35]; 4. Mathias et al. (1997) [3]; 5. http://www.wikipedia.org

#### Ion fluxes

The solute fluxes in a fluid are generally governed by diffusion, electro-diffusion (if the solute is charged) and advection [30,31,37]. Hence, we modelled the ionic fluxes in the lens using the Nernst-Plank equation with an added advection term [Eq. 3 [18,30,31,37].

(3) $$j_{\alpha }=-D_{\alpha }\nabla C_{\alpha }-Z_{\alpha }e\frac{D_{\alpha }}{k_{B}T}\nabla \phi .C_{\alpha }+u.C_{\alpha }$$

The parameters and their units are listed in [Table 1. All the ionic flow related constants utilized in this model are listed in [Table 3. The intracellular diffusion coefficients for Na^+^, K^+^ and Cl^-^ (D_i,c_) in the radial direction are estimated to be 1% of the diffusion coefficients in the cytoplasm [38]. The diffusion coefficient for small ions in the cytoplasm is assumed to be the same as in free solution. This assumption is valid if there is no significant interaction between the ions and the cell membrane or large molecules. Similarly, the extracellular diffusion coefficients for Na^+^, K^+^ and Cl^-^ (D_e,c_) are assumed to be the same as in free solution. These values are scaled by the τ_c_ [Table 3 to account for the tortuosity of the extracellular cleft. The conductivities for the Na^+^, K^+^ and Cl^-^ channels are the same as those used in the analytic model and are assumed to be spatially uniform.

**Table 3:** Solute transport properties

**Symbol**	**Description**	**Value**	**Units**
kB	Boltzmann constant	1.380 × 10^−11^	pJ/K
e	Electron charge	1.6 × 10^−10^	nC
F	Faraday constant	9.648 × 10^4^	nC/nmol
D_Na_	Free solution/cytoplasm Na^+^ diffusion^1^	1.39 × 10^−6^	mm^2^/s
D_K_	Free solution/cytoplasm K^+^ diffusion^1^	2.04 × 10^−6^	mm^2^/s
D_Cl_	Free solution/cytoplasm Cl^-^ diffusion^1^	2.12 × 10^−6^	mm^2^/s
D_Nai,c_	Intracellular Na^+^ diffusion	1.39 × 10^−8^	mm^2^/s
D_Ki,c_	Intracellular K^+^ diffusion	2.04 × 10^−8^	mm^2^/s
D_Cli,c_	Intracellular Cl^-^ diffusion	2.12 × 10^−8^	mm^2^/s
D_Nae,c_	Extracellular Na^+^ diffusion	1.39 × 10^−6^	mm^2^/s
D_Ke,c_	Extracellular K^+^ diffusion	2.04 × 10^−6^	mm^2^/s
D_Cle,c_	Extracellular Cl^-^ diffusion	2.12 × 10^−6^	mm^2^/s
g_Na_	Na^+^ fibre cell membrane conductivity^2^	2.2	mS/m^2^
g_Cl_	Cl^-^ fibre cell membrane conductivity^2^	2.2	mS/m^2^
g_K_	K^+^ surface membrane conductivity^2^	2.1	S/m^2^
Imax	Na^+^/K^+^ pump max. pump rate	0.478	A/m^2^
K_1/2Na_	Na^+^/K^+^ pump 1/2 max Na^+^ concentration	9	mM
K_1/2K_	Na^+^/K^+^ pump 1/2 max K^+^ concentration	3.9	mM

References: 1. Benedek & Villas (2000) [32]; 2) Mathias (1985) [2].

We solved these equations [Eq. 1 to Eq. 3] for the movement of ions and water in the extracellular and intracellular spaces of the lens. These two domains were linked by cell membranes which could be crossed by ions and water from one space to another [Figure 1B]. Since the water and ions enter the lens from the extracellular space, we started with these equations.

### Extracellular flux equations

The fluid flow in the extracellular clefts can be partially described by the Stokes flow equations [Eq. 1 & Eq. 2. Another part of the extracellular fluid fluxes has been shown to be due to electro-osmosis [39]. Electro-osmosis is due to the osmotic gradient created by uneven charge distribution in a fluid affected by an electric field. It has been shown that this osmosis is essential to the modelling of extracellular fluxes in the ocular lens [39].

*Extracellular fluid flows* in the lens were modelled by the Stokes equations [Eq. 1 & Eq. 2 with an added electro-osmosis term. We treated electro-osmosis as a body force term and formulated it as below for our lens model [Eq. 4 [18].

(4) $$u_{\mathrm{eo}}=\frac{\varepsilon_{r}\varepsilon_{o}\zeta }{\mu }\left(\coth\left(\frac{h}{2\lambda_{D}}\right)-\frac{2\lambda_{D}}{h}\right)\nabla \phi_{e}$$

The parameters and their units are listed in [Table 1]. We then combined this velocity (*u*_*eo*_) and the Stokes equation [Eq. 2] to drive the equation for the extracellular water velocity [Eq. 5].

(5) $$u=-\frac{h^{2}}{8\mu }\frac{dp}{dx}+\frac{\varepsilon_{r}\varepsilon_{o}\zeta }{\mu }\left(\coth\left(\frac{h}{2\lambda_{D}}\right)-\frac{2\lambda_{D}}{h}\right)\nabla \phi_{e}$$

The parameters and their units are listed in [Table 1. We didn’t consider electro-osmosis in the intracellular space portion of our model [18]. This was since the electric field gradient across cell cytoplasm was considered negligible. We then coupled the extracellular water fluxes with the ionic fluxes in this domain.

#### Extracellular ion fluxes

We computationally solved the advective Nernst-Plank equation [Eq. 3] for the lenticular extracellular ion fluxes. We modelled the coupled water and ion extracellular fluxes throughout the 3D model. These fluxes could then become trans-membranous flows at any point of the model, crossing the modelled cell membranes.

### Trans-membrane flux equations

The extracellular and intracellular spaces of this model are connected by cell membranes with specific water and ionic permeabilites. Hence the water could flow cross fibre cell membrane between the extracellular and the intracellular spaces. To represent these transmembrane fluxes in our model, we treated the fibre cell membrane as a semi-permeable membrane [2,7,10,16] through which fluid passes due to a combination of hydrostatic and osmotic pressure gradients [19,40,41]. The velocity of the water flowing through the membrane was then governed by the following equation [Eq. 6 [18].

(6) $$u_{m}=-L_{P}\Delta p-\sigma \;L_{P}\;\text{R}T\Delta \;\text{Os}$$

The parameters and their units are listed in [Table 1]. We calculated the hydraulic and osmotic pressure gradients, *Δp* and *ΔOs*, from the pressure differences on two sides of the membrane. We estimated the general osmolarity, *Os*, of the intracellular or extracellular spaces from the local sum of all the modelled ions (i.e. Na^+^, K^+^ and Cl^-^).

(7) $$\text{Os}=\sum_{\alpha }C_{\alpha }$$

As mentioned previously, we modelled the ion and water fluxes to go hand-in-hand throughout our computational platform. Hence, the modelled ions (i.e. Na^+^, K^+^ and Cl^-^) accompanied the trans-membrane water fluxes into the cells, utilizing the membrane ionic channels. The lenticular membrane conductivity for each modelled ion had been calculated based on experimental data [8,11,42,43]. We used similar values for membrane conductivities for various modelled trans-membrane ion fluxes. We implemented these trans-membranous ionic flows using the following equations [Eq. 8 to Eq. 10 [18].

(8) $$j_{\alpha }=\frac{\text{ga}}{F}\left(V_{m}-E_{\alpha }\right)$$

(9) $$E_{\alpha }=-\frac{k_{B}T}{Z_{\alpha }e}\text{ln}\left(\frac{C_{\alpha 2}}{C_{\alpha 1}}\right)$$

(10) $$V_{m}=\phi_{i-}\phi_{e}$$

The parameters and their units are listed in [Table 1]. In the above equations, *E* is the Nernst potential. At the Nernst potential, there is no net flow of ions across the cell membrane through the channels. Hence, in our model we linked the trans-membrane water and ion fluxes by ionic concentrations (i.e. osmosis) and membrane potential (i.e. Nernst potential). After crossing the membrane, we treated the water and ion fluxes as intracellular fluxes.

### Intracellular flux equations

We modelled the fluxes in the intracellular space to pass through a mixture of cells cytoplasm and membranes [44]. This was due to the large size of each element in our model compared to the lens cell volumes. It was impractical to discretely model the intracellular flow through each cell cytoplasm and membrane in a given element. Instead, we homogenized the flow equations to obtain one formula, describing the net cytoplasmic and membranous fluxes through every element.

To homogenize the flow equations, we assumed the intracellular fluid fluxes are primarily restricted by the cell membranes. We made this assumption since it was shown that diffusivity between cells is about on hundred times lower than in the cytoplasm [44]. We also assumed negligible hydraulic and osmotic pressure gradients across the cytoplasm of a single cell. We considered these fields to vary only among neighbouring elements, across the modelled membranes. Hence we assumed the pressure fields as step-function changes across the cell membranes. We therefore implemented the intracellular fluid flow to be driven by the pressure and concentration step-changes across the cell membranes. We calculated the intracellular fluid velocities using the following equation [Eq. 11.

(11) $$u_{i}=L_{p}\left(-\Delta p_{i}-\sigma_{i}RT\;\Delta Os_{i}\right)$$

The parameters and their units are listed in [Table 1. Since the implemented intracellular water fluxes were accompanied by the ionic fluxes in our model, we assumed the intracellular ionic fluxes to be governed by the advective-Nernst-Plank equation [Eq. 3. As mentioned above, every element of the intracellular space in our model was treated as a homogenised mixture of cytoplasm and membranes. In reality a network of gap junctions forms cell-to-cell channels that connect the cytoplasms of adjacent cells [40,45]. We derived the ionic flux equation for a single gap junction channel [Eq. 3 and then integrated this equation over a network of pores in a given cell membrane [18]. We used [Eq. 3 for ionic fluxes, assuming free movement of ions through each pore. In this case we could assume the diffusion coefficient (*D*) in the pore to be equivalent to the cell’s interior diffusion coefficient. We also assumed the ion concentrations (*C*) and electric potentials (*ϕ*) to vary linearly between the opposite openings of each pore. Therefore, we could approximate these variables over the length of the pore (*x*), by the following equations [Eq. 12 & Eq. 13.

(12) $$\frac{dC_{\alpha }}{dx}=\frac{C_{\alpha ,2}-C_{\alpha ,1}}{\Delta x}=\frac{\Delta C_{\alpha }}{\Delta x}$$

(13) $$\frac{d\phi_{\alpha }}{dx}=\frac{\phi_{2}-\phi_{1}}{\Delta x}=\frac{\Delta \phi }{\Delta x}$$

Here, the subscripts *1* and *2* referred to the inlet and outlet of the modelled pore. We approximated the ion concentration in the pore (*C*_*α*_) by the mean concentration [Eq. 14].

(14) $$C_{\alpha }=\frac{C_{\alpha ,1}+C_{\alpha ,2}}{2}$$

By substituting the above equations [Eq. 12 - Eq. 14] in the advective-Nernst-Plank formula [Eq. 3], we derived the following form [Eq. 15].

(15) $$j_{\alpha }=-D_{\alpha }\nabla C_{\alpha }-z_{\alpha }e\frac{D_{\alpha }}{k_{B}T}\nabla \phi .C_{\alpha }+u.C_{\alpha }$$

The subscript *α* indicated the solute species and the rest of the variables are listed in [Table 1]. The diffusion coefficient (*D*) in the above equation is mainly set by the directional conductivity of the gap junctions [Eq. 16].

(16) $$D_{\alpha }=\frac{R.T.G_{\alpha }}{F^{2}.C_{\alpha }}$$

The parameters and their units are listed in [Table 1. In this implementation, the diffusivity of a gap junction pore (*D*_*α*_) is related to its conductivity (*G*_*α*_). Based on experimental observations, the measured conductivities of the mouse lens have been assorted into two main regions. The inner 85% of the lens is anucleated [1] and has an approximately spatially uniform distribution of gap junctions. For this inner part of the lens a homogeneous value of conductivity (*G* = 0.17mS/cm) has been used [7]. The outer 15% of the lens on the other hand is nucleated [1]. The radial conductivity, G_r_ has been assumed to vary depending on the angular location (*ϕ*) and the conductivity of the gap junctions [Eq. 17 [7].

(17) $$G_{r}=G_{\max}.\text{co}s^{2}\phi$$

Here *ϕ* is the angular degree from the equator (i.e. *ϕ* = 0° at equator, *ϕ* = 90° at anterior pole and *ϕ* = −90° at posterior pole). It has been determined that *G*_*max*_ = 0.6mS/cm in the radial orientation, and in the other directions *G* = 0.23mS/cm [7]. We modelled the intracellular ionic fluxes to be directed by the above equations and the regional distribution of gap junctions [Eq. 15 - Eq. 17. According to the microcirculation theory, these ionic fluxes then moved towards the periphery of the lens accompanied by water flows until they reach the boundary of the model.

### Surface flux equations

Once intracellular fluxes reach the boundary of our 3D computational mesh they are treated as surface fluxes. We modelled the periphery of the lens to be similar to previously implemented cell membranes. The only difference was that at this point we had to consider the surrounding boundary conditions that are determined by the ionic composition of the bathing media in the system of equations. At the surface of the lens, fluid is transported between the intracellular space and the outside. We modelled these surface fluid flows as simple trans-membrane fluxes and calculated their velocities using the equation [Eq. 18].

(18) $$u_{s}=-L_{s}\left(p_{o}-p_{i}\right)-\sigma_{s}RT\;L_{s}\left(Os_{o}-Os_{i}\right)$$

The parameters and their units are listed in [Table 1] and the subscript “*o”* points to the outside (i.e. boundary) conditions. We considered the surface fluxes as the output of the system, while the extracellular fluxes were its input. For an incompressible stable system, like the current lens model, the input and output levels should equate at all times and this conservation of mass was controlled for as part of the Stokes equations [Eq. 1].

At the surface of the lens, we modelled the intracellular space to be coupled to the outside media. We created this coupling by a network of K^+^ channels and Na^+^/K^+^-pumps in the surface membranes [39,44]. To model ionic fluxes through the K^+^ channel, equations [Eq. 8 to Eq. 10 were used. To implement the Na^+^/K^+^-pumps at the surface of the lens we used a previously published approach [39] that utilised the following set of equations [Eq. 19 to Eq. 21 to model these pump rate.

(19) $$Ip=I_{\max}{\left(\frac{C_{Na_{i}}}{C_{Na_{i}}+K_{\mathrm{Na}}}\right)}^{3}.{\left(\frac{C_{K_{o}}}{C_{K_{o}}+K_{K}}\right)}^{2}$$

(20) $$K_{\text{Na}}=\left(\sqrt[{3}]{2}-1\right)K_{0.5\text{Na}}$$

(21) $$K_{K}=\left(\sqrt{2}-1\right)K_{0.5K}$$

Here, *I*_*max*_ was the maximum current rates through the pumps. The parameters and their units are listed in [Table 1. The *K*_*0.5Na*_ and *K*_*0.5K*_ were the concentrations of Na^+^ and K^+^ when the pump’s current was half the maximum current [39]. We also implemented the known circumferentially-varying distribution of ionic pumps around the lens [7]. The ionic pumps of the lens are more abundant around its equatorial area and scarce around the polar regions [7,39]. It has been suggested that the currents produced by Na^+^/K^+^ ATPase pumps around the ocular lens are governed by [Eq. 22 [7].

(22) $$I_{p}=I_{p-\text{min}}+\left(I_{p-\text{max}}-I_{p-\text{min}}\right).cos^{2}\phi$$

Here *ϕ* is the angular degree from the equator (i.e. *ϕ* = 0° at equator, *ϕ* = 90° at anterior pole and *ϕ* = −90° at posterior pole). Experiments have shown that *I*_*p-min*_ = 4 μA/cm^2^[39] around the poles and *I*_*p-max*_ = 26 μA/cm^2^ near the equator [41]. Hence, at this point the model included the regional distribution of the ionic pumps. We then calculated the surface Na^+^ and K^+^ fluxes by using the pump rate (*I*_*p*_) estimated above. We estimated these pump-generated molar fluxes at every point on the surface of the 3D mesh [Eq. 23 & Eq. 24.

(23) $$j_{Na,p}=3\frac{\text{Ip}}{F}$$

(24) $$j_{K,p}=-2\frac{\text{Ip}}{F}$$

We modelled the total surface ionic fluxes by combining the pump-generated and channel-based flows [Eq. 8, Eq. 23 & Eq. 24].

### Electro-neutrality equation

Being a closed system, the model operates in the condition that at every point of time electro-neutrality is preserved. In this model we used a weak form of electro-neutrality [Eq. 25] to enforce this constraint.

(25) $$\sum_{\alpha }Z_{\alpha }\frac{\text{dC}\alpha }{\text{dt}}=0$$

We assumed the initial condition of the system to be electrically neutral. Hence, the equation above ensures the electro-neutrality of the model at any point of time. We applied this equation over all the ion species modelled here. This was based on the assumption that the ion species not modelled (e.g. calcium) had no significant influence on electro-neutrality of the model.

### Solving the model

**Figure 2:**
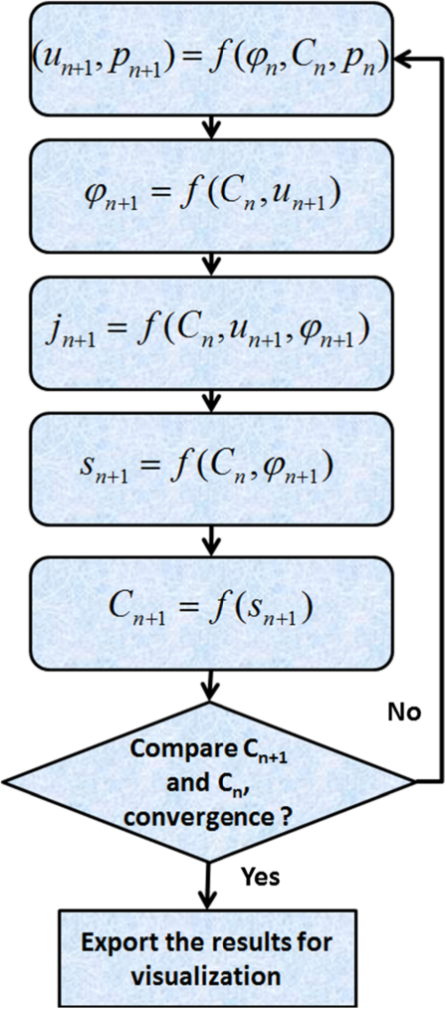
**Steps of the iteration solution of the model reviewed in the text.** The first step solved the Stokes equations, using the initial conditions (*C*_*0*_*, ϕ*_*0*_) and boundary conditions (*C*_*αo*_*, ϕ*_*o*_*, p*_*o*_), to calculate the fluid velocity (*u*_*n+1*_) and pressure fields (*p*_*n+1*_). The second step used electro-neutrality to calculate new potential fields (*ϕ*_*n+1*_). Step three calculated new solute fluxes (*j*_*n+1*_) using current concentration fields (*C*_*n*_), new potential field (*ϕ*_*n+1*_) and new velocity field (*u*_*n+1*_). The forth step used current concentration fields (*C*_*n*_) and new potential field (*ϕ*_*n+1*_) to calculate the new trans-membrane or surface solute sources (*s*_*n+1*_). Step five involved using the newly calculated solute sources (*s*_*n+1*_) to calculate new concentration fields (*C*_*n+1*_). The model then checked for the convergence criterion automatically and updated the initial fields and repeated these steps if the criterion was not met.

We implemented all of the mentioned water and ion flux equations in our model. This was in order to create an interlinked system of fluid dynamics of the ocular lens. The implemented system of equations is summarized in the following figure [Figure 1B. To solve this system of equations we used an adaptive Euler method, used to achieve a converged steady state solution [18]. Each iteration of the adaptive Euler method involved several steps to solve the coupled transport equations, followed by a solution update step [Figure 2. The model began with a representative mesh of the lens, the initial conditions (*C*_*0*_*, ϕ*_*0*_) and boundary conditions (*C*_*αo*_*, ϕ*_*o*_*, p*_*o*_). Ideally, one would start with initial concentration fields that are close to the expected final concentration fields.

### Finite Element Mesh Creation

**Figure 3:**
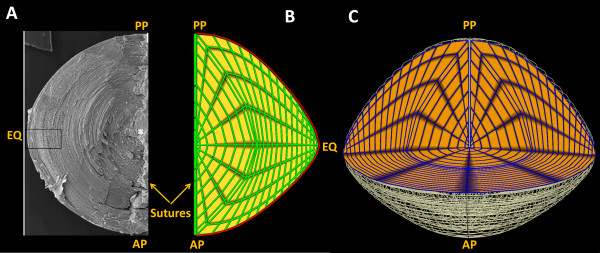
**Comparison of lens fibre cell geometry and its finite element mesh representation.** A: A scanning electron microscope image of a mouse lens reproduced from [46]. B: 2D projection of half of the finite element mesh generated used to model the mouse lens. The outer surface of the lens, coloured in red, is where the computational boundary conditions are applied to the mesh. C: Example of a quarter section of the 3D model, used in the Figure 4 and Figure 5 to view standing electrochemical fields predicted by model. The anterior pole (AP), posterior pole (PP) and equator (EQ) are labelled as points of reference.

Since a wealth of experimental data exists on the mouse lens we chose it to create an anatomically accurate scaffold to implement our modelling approach. The mouse lens has an equatorial radius of 0.125 cm, posterior thickness of 0.1 cm and anterior thickness of 0.085 cm [42]. We used a cylindrical polar coordinate system (r, θ, z) and the Cubic Hermite basis function to create a smooth 3D computational mesh of the mouse lens [Figure 3 and Table 4. When a biological tissue is modelled as a macroscopic continuum, elements in the representing mesh are much larger than the cells. In our model, the intra- and extracellular spaces of the lens therefore could not be represented disjointedly. Hence, we modelled that these spaces, while mathematically distinct, coexist in the same mesh element and the cell membranes are evenly spread all through. Every element in our model thus represented a cluster of many fibre cells and enclosed extracellular space. This method of “bidomain” modelling has been well-explained previously [43].

**Table 4:** Finite element mesh specifications for the current model

**Modelling property**	**Property value**
Coordinate system	Cylindrical Polar
Basis function	Cubic Hermit
Number of nodes	5281
Number of elements	2130

### Boundary conditions

Since our model is solved using an iterative method, it was important to choose appropriate initial boundary conditions. In previous microcirculation models, the boundary conditions have been chosen to be ionic concentration at the surface of the lens (*C*_*Na-io*_*, C*_*K-io*_*, C*_*Cl-io*_*, C*_*Na-eo*_*, C*_*K-eo*_*, C*_*Cl-eo*_) [3,10]. Here subscript “o” denotes the surface of the lens. We adopted the same approach to solve our 3D model. Boundary conditions were taken from the measurements of ionic concentrations from the surface of ocular lenses in different species [26-28,47] and are listed in [Table 5. Using these initial conditions we solved the model and assumed the initial extracellular electric-potential and hydraulic pressures to be zero. These fields were then calculated during the first iteration round and substituted in the model for the following iteration cycle with this iteration being repeated until convergence was reached.

**Table 5:** Initial conditions at outer lens boundary for the present model

**Species**	**Description**	**Quantity**
Na_e_ (mM)	Extracellular Na^+^ concentration	110
K_e_ (mM)	Extracellular K^+^ concentration	8
Cl_e_ (mM)	Extracellular Cl^-^ concentration	115
Na_i_ (mM)	Intracellular Na^+^ concentration	7
K_i_ (mM)	Intracellular K^+^ concentration	100
Cl_i_ (mM)	Intracellular Cl^-^ concentration	10
T (K)	Temperature	310

### Convergence criterion

We defined the maximum change of concentrations between two consecutive iterations (*C*_*n+1*_*- C*_*n*_) in all the elements as the convergence parameter. We observed that using the above boundary and initial conditions, this error was decreased with each iteration cycle. We set the model’s convergence criterion to be less than 5 mM. We believed that level of iteration error was adequate, since most of the solution fluctuations were caused by [*K*^*+*^]_*i*_ and [*Na*^*+*^]_*e*_ fields and the enforced iteration error threshold was less than 5% of the initial field value.

We solved the 3D model on our high performance computer (HPC) at the Auckland Bioengineering Institute (ABI). Our mainframe was comprised of an IBM server with 64 processors of 1.9 GHz calculation speed, peak performance of 306.21Gfps and 256 GB of physical memory.

### Displaying the data

Text formatted file of the model’s computed fields were linked via JAVA programming language format to CMGUI ( http://www.cmgui.org) [48,49], an advanced 3D visualisation software package with modelling capabilities. CMGUI was used for model visualisation and manipulation and allowed for automated the scaling and false colouring of the data. The following presentations were all created using this method.

## Results

Using the boundary conditions listed in [Table 5], we solved the model and generated 3D maps of standing fields of intracellular and extracellular ion concentrations, electrical potentials and pressure, plus circulating ionic and water fluxes. In this section we first use quarter section views of the 3D model [Figure 3C] to represent regional differences in standing electrochemical and pressure fields, allowing these predicted properties to be compared to experimentally derived values. Then we use the full model view to generate 3D vector maps that visualize the predicted ion and water fluxes in the lens for the first time.

### Standing electrochemical and pressure fields

**Figure 4:**
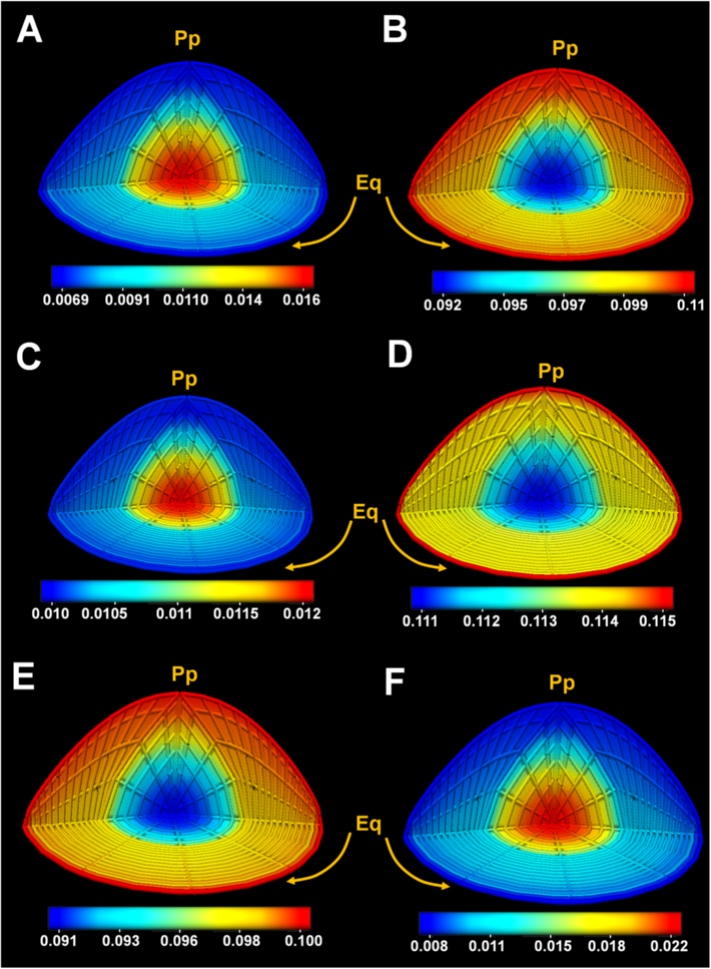
**Standing ionic concentration fields modelled in the mouse lens.** 3D quarter section views of the standing concentration fields calculated from the model for the intracellular (A, C, E) and extracellular (B, D, F) space for Na^+^ (A, B), Cl^-^ (C, D) and K^+^ (E, F). Note that in the extracellular space, a diffusive-conductive gradient was created by the decreasing concentration of Na^+^ from the periphery towards the centre. In the intracellular space, however, the concentration field for Na^+^ was reversed. The small extra- and intracellular Cl^-^ concentration field in the radial direction is close to its predicted steady state [3]. K^+^ is transported into cells mostly close to the periphery of the ocular lens [16] and so its high concentration at the intracellular space of the surface and accumulation in the core of the extracellular space. The values on the colour-bars are in Molar units.

The operation of the microcirculation system will at steady state create standing fields in ion concentrations and membrane potentials in both the extracellular and intracellular compartments. The 3D representations of the ion concentration fields for Na^+^, K^+^ and Cl^-^ in both compartments are illustrated in Figure 4. The values for these concentrations were then extracted and presented in Table 6. To assess the accuracy of the standing ionic gradients produced by the model, we wanted to compare them to existing experimental data. Unfortunately no data on the concentration of ions in the extracellular space of the lens is available. However, ion concentrations in the intracellular space have been measured using a variety of techniques. Wang et al. [50] have used ion selective micro-electrodes to show a Na^+^ concentration gradient exists in the mouse lens, the magnitude of which is predicted by our model [Table 6. Average concentrations of Na^+^, K^+^ and Cl^-^ in the lens have also been measured using chemical analysis in a number of species [26-28,47]. To facilitate comparison between these previous measurements of total lens ion concentrations and the values predicted by our model, we extracted the mean concentration from our calculated ion fields [Table 6. Here we observed a good compatibility between the modelled and measured values.

**Table 6:** Comparison of modelled standing electrochemical gradients outcomes and the existing empirical data

**Standing fields**	**3D model radial range**	**Experimental radial range**	**3D model averaged**	Experimental averaged^*^
[Na^+^]_i_ (mMol)	6.9 to 16	5 to 16 ^1^	12.5	13.8 ^3^, 21.5 ^4^
[K^+^]_i_ (mMol)	91 to 100	Not Measured	95	91.2 ^5^
[Cl^-^]_i_ (mMol)	10 to 12	Not Measured	11.35	16.8 ^5^, 12.3 ^5^
ϕ_i_ (mV)	−57 to −64	−50 to −70 ^2^	−61.5	−70 ^6^
ϕ_e_ (mV)	0.0 to −27	0 to −36 ^2^	−13.5	−30 ^6^
P_i_ (kPa)	0.0 to 10	0.0 to 48 ^7^	4.8	Not Measured

References: 1- (Wang et al. 2004) [50]; 2- (Mathias and Rae 1985) [16]; 3- (Delamere and Duncan 1977) [27]; 4- (Duncan 1970) [47]; 5- (Guerschanik et al. 1977) [28]; 6- (Delamere and Paterson 1979) [51]; 7- (Gao et al. 2011) [29].

**Figure 5:**
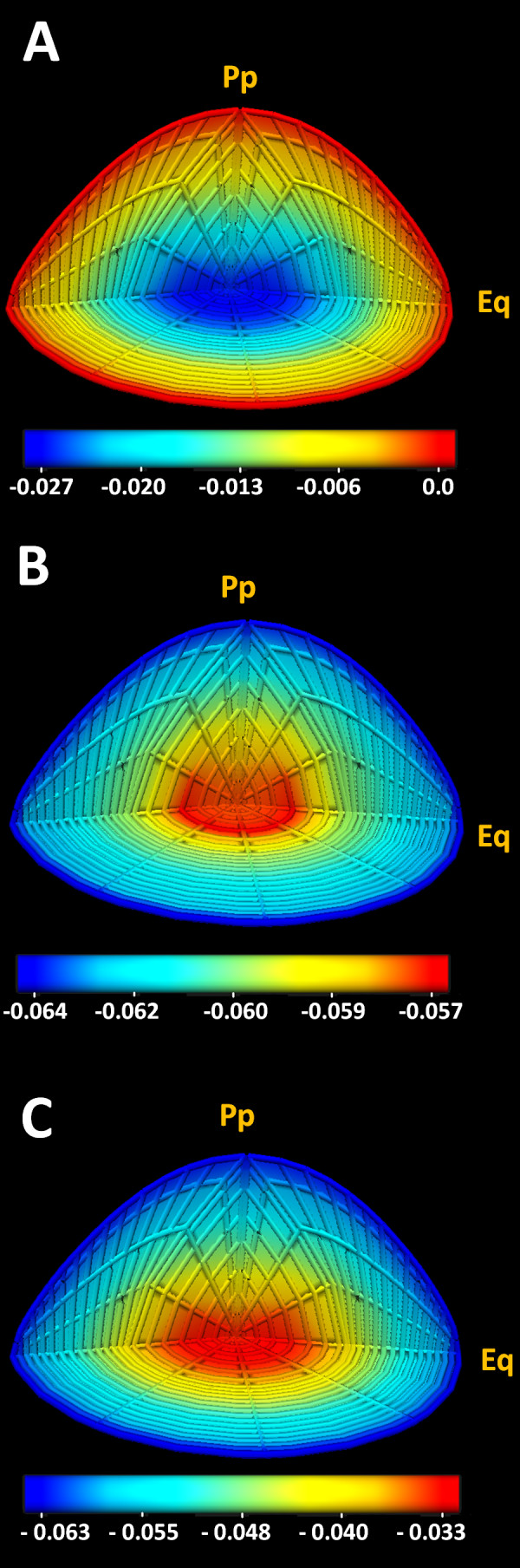
**Standing electrical fields modelled in the mouse lens.** 3D quarter section views of the standing electrical fields calculated from the model for the extracellular (A) and intracellular (B) space plus the transmembrane potential field (C). Both the extracellular and intracellular electrical fields decrease in value from surface to centre, but the magnitude of the observed change in the intracellular potential is smaller due to the high level of gap junction coupling between inner-cells and surface cells. The trans-membrane electrical potential was calculated as the difference between the intracellular and extracellular potentials. This field appeared to be decreasing in magnitude from the surface towards the core of the lens. The values on the colour-bars are in Volt units.

The trans-membrane ionic concentration fields determined by the model were then used to calculate the intra- and extracellular and trans-membrane potential fields [Figure 5. These calculated values agreed with lens potentials measured experimentally [9,51], [Table 6. The model predicted a decrease in extracellular space electrical potential from the periphery to the core. This is thought to be indicative of an increase of extracellular space resistance with depth into the lens [8]. In contrast, a smaller change in the intracellular electric potential field was predicted by the model. This small change has been observed previously [2,10] and has been attributed to a high level of gap junction coupling between inner-cells and surface cells containing K^+^ channel and pumps that dominate the lens potential [15]. This meant that the potential between the extracellular and intracellular spaces depolarized from -63 mV at the surface to -33 mV in the centre of the model [Figure 5C. These changes in potential fields with distance into the lens were consistent with the movement of a positively-charged ion like Na^+^, into the lens via the extracellular space, down its electrochemical gradient across the cell membrane and outwards in the intracellular space.

**Figure 6:**
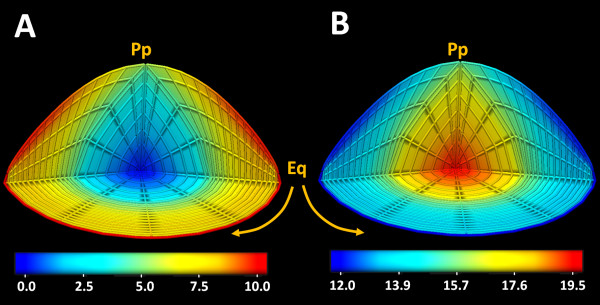
**Hydrostatic pressure fields calculated by the mouse lens model.** 3D quarter section views of the standing hydrostatic pressure fields calculated for the intracellular (A) and extracellular (B) spaces. The inwardly gradient of extracellular pressure field indicated the direction of the fluid flow towards the centre of the lens model. On the other hand, the gradient of the estimated intracellular pressure field indicates outward fluid flow in this compartment of the lens. The values on the colour-bars are in kPa units.

The Stokes equations [Eq. 1 & Eq. 2 used to model the fluid dynamics of the lens has an inherent pressure term, which allows us to calculate the standing fields of hydrostatic pressure within the intra and extracellular compartments [Figure 6. The model calculated an extracellular pressure gradient that was highest at the periphery and lowest in the core [Figure 6A and an intracellular hydrostatic pressure gradient that was lowest in the periphery and highest in the core [Figure 6B. Recently the existence of an intracellular pressure gradient has been confirmed experimentally [29]. A comparison of the calculated and measured intracellular pressure fields indicated that the orientations of the gradients are similar but the magnitudes differ by a factor of ~4.5 [Table 6. This disagreement is discussed further in the Discussion.

**Figure 7:**
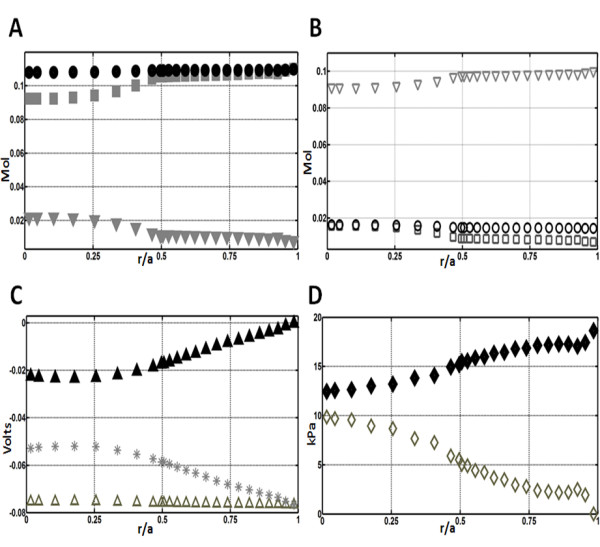
**Comparison of the calculated standing fields generated by the model.** To facilitate the comparison of the calculated standing fields, parameters are extracted from the equatorial radius of the model and plotted against the normalized distance from the centre (r/a). (A) Plot of the extracellular Na^+^(■), Cl^-^ (●) and K^+^ (▼) concentrations (Molar units) versus relative distance from the lens core (r/a). (B) Plot of the intracellular Na^+^(□), Cl^-^ (○) and K^+^ (∇) concentrations (Molar units) versus relative distance from the lens core (r/a). (C) Plot of the intracellular (Δ), extracellular (▴) and the trans-membrane (*) electrical potential fields (Volts units) versus relative distance from the lens core (r/a). (D) Plot of the intracellular (◊) and extracellular (♦) hydrostatic pressure fields (kPa units) versus relative distance from the lens core (r/a). The electrochemical and pressure gradients are oriented to direct the extracellular solute and fluid fluxes towards the core of the lens, while fields in the intracellular space appear to favour an outward flow.

The results obtained from our 3D model of the calculated electrochemical and pressure fields can be simplified to facilitate comparison between parameters, by extracting values from a defined equatorial axis and plotting them against normalized distance from the centre (r/a) [Figure 7]. In the lens, these fields give rise to the circulating fluxes modelled below.

### Circulating fluxes

**Figure 8:**
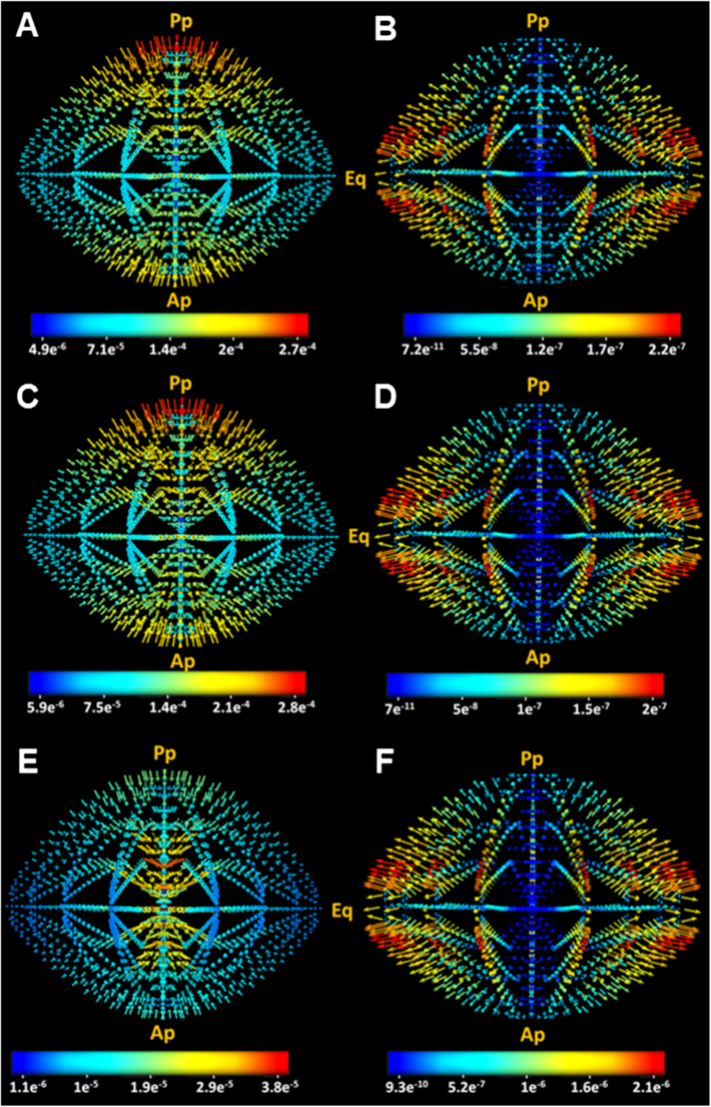
**Circulating ionic currents modelled in the mouse lens.** 3D vector maps of circulating ionic currents calculated from the model for the extracellular (A, C, E) and intracellular (B, D, F) space for Na^+^ (A, B), Cl^-^ (C, D) and K^+^ (E, F). Extracellular Na^+^ fluxes seemed to be largest of the ionic fluxes with a maximum located around the polar regions of the model. The intracellular fluxes on the hand were outwardly and equatorially oriented. Extra and intracellular Cl^-^ fluxes appeared to follow the same path into the lens with close magnitudes to Na^+^ fluxes in the same compartment. The K^+^ extracellular fluxes seemed to be negligible compared to other ions, while intracellular values were the largest and highly concentrated around the equatorial region of the model. The numbers on the colour-bar are in (mMol/cm^2^.s) units.

We calculated extracellular and intracellular ionic fluxes throughout the lens using the advective Nernst-Plank equation. Extracellular space fluxes of Na^+^ were directed into the lens and had the highest magnitude at the poles [Figure 8A [4]. In contrast, intracellular space fluxes of Na^+^ were directed out of the lens and had their highest magnitude at the equator [Figure 8B. Extracellular [Figure 8C and intracellular [Figure 8D fluxes of Cl^-^ appeared to mirror the Na^+^ fluxes as would be expected to preserve electro-neutrality. Relative to extracellular fluxes of Na^+^ and Cl^-^, the calculated extracellular K^+^ fluxes were of reduced magnitude [Figure 8E. However the intracellular space K^+^ fluxes were of larger magnitude and were concentrated at the equator [Figure 8F, consistent with the abundance of pumps and K^+^ channels known to be localized in this region of the lens [3,10,16].

Although ionic fluxes within the lens have yet to be determined experimentally, measurements of net current densities at the lens surface are available [5,41]. To facilitate a comparison between these measurements and our model’s predictions, we calculated net current densities using [Eq. 26 & Eq. 27.

(26) $$J=\left(j_{\text{Na}}+j_{K}-j_{\mathrm{Cl}}\right).F$$

(27) $$j=\frac{j_{i}.A_{i}+j_{e}.A_{e}}{A_{i}+A_{e}}$$

**Figure 9:**
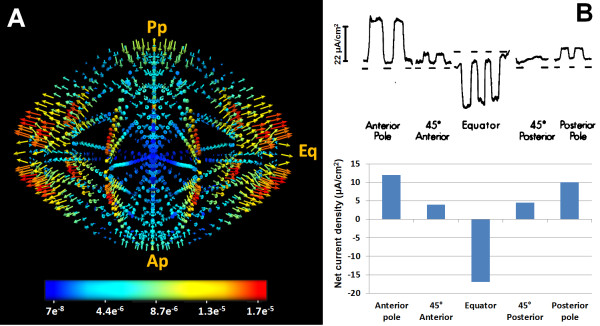
**Net current densities in the lens.** A: 3D vector map of net current densities calculated by the model. The net current densities were clearly inwardly around the anterior and posterior poles and outward around the equatorial plane of the model. B: Surface net current densities measured (upper panel) using the vibrating probe technique around the lens [5], compared to surface values extracted from the 3D model and plotted (lower panel). These panel show the agreement of modelled the surface currents with the experimentally measured values. The numbers on the colour-bar are in A/cm^2^ units.

The parameters and their units are listed in [Table 1. The resultant 3D map of the current densities throughout the lens are shown in Figure 9A, and the values extracted for the surface densities located at both poles and the equator are listed in Table 7. Our model clearly shows a net current influx at the poles and a net efflux at the equator [Figure 9B, a pattern in agreement both in direction and magnitude with previous electrophysiological measurements conducted on a variety of lenses [Table 7, [5,41]. What our model also showed for the first time was the pattern in magnitude of net current fluxes within the lens.

**Table 7:** Comparison of modelled circulating currents and the existing experimental mouse data

**Parameter**	**Model prediction**	**Experimental data**
Net current density (μA/cm^2^)		
J_equator_	17, outward	22 ^1^, 20 ± 2.6 ^2^, outward
J_anterior pole_	12, inward	26 ^1^, 32 ^2^, inward
J_posterior pole_	10, inward	7 ^1^, 42 ^2^, inward
Fluid velocity (nm/s)		
V_equator – intracellular_	2.1, outward	Not Measured
V_anterior pole – extracellular_	190, inward	Not Measured
V_posterior pole – extracellular_	250, inward	Not Measured

References: 1- (Robinson and Patterson 1982) [5]; 2- (Parmelee 1986) [41].

**Figure 10:**
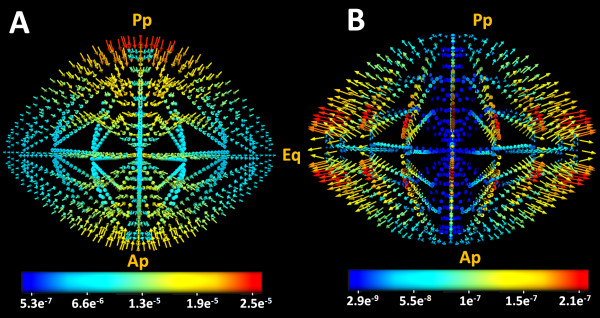
**Circulating fluid velocity fields modelled in the mouse lens.** 3D vector maps of circulating fluid velocities calculated from the model for the extracellular (A) and intracellular (B) space. The maximum fluid velocity in the extracellular space was inward at the anterior and posterior poles and the magnitude of the extracellular fluid velocity dropped towards the equatorial region. Conversely, the intracellular fluid velocity fields were predicted to be outward, suggesting the exit of the fluid via intracellular space throughout the lens; with the peak of intracellular velocity calculated to occur in the equatorial region of the lens. The numbers on the colour-bar are in cm/s units.

The calculated ionic current densities however, are only one leg of the microcirculation system. To study the water fluxes generated by ion flow, we used the Stokes equations to calculate water velocities in the intracellular and extracellular spaces of our model [Figure 10. We observed that the extracellular fluid velocities were inward and maximal at the poles [Figure 10A. Conversely, the intracellular fluid velocities were outward and maximal at the equator [Figure 10B. Thus our model generates the theoretical water fluxes first proposed by Mathias et al. [16], but unfortunately no experimental data currently exists to verify the accuracy of the calculated magnitudes [Table 7.

## Discussion

It has been proposed that the circulating currents observed experimentally in lenses from a variety of species [5,14,41,52] drive an internal microcirculation system. In the absence of a blood supply the microcirculation delivers nutrients and removes metabolic waste products from inner fibre cells, while maintaining ionic homeostasis [7]. The circulating fluxes are thought to be the net result of spatial differences in the location of ion channels and transporters that determine local membrane permeability, and the density of gap junction channels that direct intercellular fluxes within the lens [3]. We first created a 3D mesh of the lens structure. We then implemented a series of equations that describe the local transport properties of cells in different regions in the lens. Finally we solved these equations using a FEM approach at each location of the model. In summary, we have produced a computer model of the lens that not only accurately predicts standing ionic concentrations [Figure 4 and electrical [Figure 5 gradients and the existence of a pressure gradient [Figure 6, but also produces the first 3D vector maps of predicted current [Figure 8 and fluid [Figure 10 fluxes inside the lens. The observed agreement between experimentally measured values and those calculated by our model [Table 6 & Table 7, suggest that our computer model is mimicking lens physiology and generates a microcirculation. However, the underestimation of the magnitude of the intracellular pressure gradient highlights the fact that the model is only as robust as its underlying assumptions and will require additional refinement and revision as new experimental data on lens structure and function becomes available. In the following sections we discuss the assumptions and limitations of our current model with the view to highlight areas where further refinements of the model are required.

The solute flux in a fluid is governed by diffusion, electro-diffusion (if the solute is charged) and advection. Diffusion is the random walk of particles due to Brownian motion. Electro-diffusion is the flux of a charged particle due to the force applied by an electric field. Advection is the transport of a solute by a fluid that is moving. The physical origins of these transport processes and the derivations of the equations have been discussed previously in the literature [31,32]. The implementation of these equations in a computational platform is very well explained elsewhere. In this study, we implemented the driven equations and solved for a set of converged-upon 3D fields. This method produced the calculated standing ionic concentration gradients in the lens predicted by our model [Figure 4.These gradients in turn gave rise to a trans-membrane electric-potential gradient field [Figure 5, based on the Hodgkin–Huxley model. Consider a cell membrane that is not equally permeable to all the present ionic species on either side of it. Here, the permeable ions will tend to move down their concentration gradient taking their electrical charge with them as they go. Therefore, an electrical potential will be generated, which will drive them in the reverse direction. An electrochemical equilibrium will be reached when the diffusive force equals the electromotive force.

Our model has calculated a hydrostatic pressure gradient [Figure 6, the existence of which has recently been confirmed experimentally [29]. While the orientations of the calculated and measured pressure gradients are similar, they differ in magnitude (48kPa compared to 10kPa). It has been proposed that this pressure gradient is generated by the restricted flow of water from the centre to the periphery of the lens by a pathway mediated by gap junction channels; since genetic manipulations to increase or decrease gap junction numbers produces inverse changes in the pressure gradient. This illustrates that structural components of the lens can influence the magnitude of the pressure gradient. This suggests that the difference between calculated and measured pressure fields may reflect the absence of a structural feature not currently captured in our model. In this regard we have recently identified a zone in the inner cortex of the lens that exhibits a reduction in the penetration of solutes and water [19,22] that could influence the magnitude of the calculated pressure gradient. Future updates of the model will include such newly discovered structural elements, allowing their effect on the calculated pressure fields to be assessed.

The electrochemical fields, combined with the hydrostatic pressure gradients [Figure 6 in our model generate the circulating ionic currents throughout the lens [Figure 8 & Figure 9. The ionic fluxes are accompanied by water flows in the microcirculation system. The water fluxes are generally described by the Navier–Stokes equations, which are derived from the conservation of mass, momentum, and energy principals. Also, the fluid flow in the lens can be described as slow or low-Reynolds number flow. It is also reasonable to assume that the fluid flow in a normal lens is near or at steady-state at all times [18]. These simplifications reduce the Navier–Stokes equations to the Stokes equations. The derivation and implementation of these equations into a computational framework is discussed elsewhere [16,18]. In our model, solving this set of equations results in the calculation of the water flow velocity fields [Figure 10. Although the current model appears to accurately mimic the physiological homeostasis of the lens [Table 6 & Table 7, there are still some aspects that need future improvement.

Our model presently has been implemented with a line suture structure which runs from the anterior to the posterior of the lens, through its core [Figure 3A&B]. However the mouse has a Y-shaped suture that rotates 180 °C from the anterior to the posterior pole [42]. Hence, we are planning to improve the current model with an anatomically accurate asymmetrical 3D structure of the sutures. In our current model, the 3D extracellular solute diffusion coefficients are constant throughout. However, we [21,53] and others [54-56] have recently identified a barrier to the movement of solutes in the lens, using variety of techniques. We strongly believe that the existence of this barrier is very important in shaping the fluid dynamics of the lens and in general its microcirculation. Hence, we will implement the imaged barrier as a part of continuous improvement of our computational model.

Another important structural feature of the ocular lens is the presence of a Gradient of Refractive Index (GRIN), which acts to correct for inherent spherical aberration to improve the optical properties of the lens [57]. This GRIN profile of the lens is directly dependant on the local water/protein concentration makeup of the lens [58,59]. Recently, we have experimentally showed that the water/protein gradient of the lens is actively upheld by the microcirculation system [19]. Hence, another step in the improvement of the current model is to add equations to estimate the concentration gradient of water in the lens. Using that calculated field, we will be able to estimate the 3D GRIN maps of the lens. We would then use these 3D GRIN maps and optical ray-tracing software to produce a model that links lens physiology to the optical properties of the lens.

Our first generation 3D finite element model of lens structure and function describes ion and fluid dynamics in the mouse lens. We chose to model the mouse lens as ion and fluid dynamics have been extensively studied in this species [3,4,15,16]. We also believe the model is an essential first step towards creating a comprehensive model of the human lens. Any model of the human lens model would need to include its more complex structural features and would need to be created so that its dimensions could be altered to study the effects of lens growth and ageing on the circulation system [60]. This future model would enable us to study the changes in lens physiology thought to underlie the initiation of age related nuclear cataract.

## Conclusion

During this project, a 3D model of the flux movements inside the mouse ocular lens was designed and executed using our high performance computer (HPC). Reviewing the results of the current model, it appears that solute fluxes, accompanied by water, enter the lens via the extracellular space all around it but with larger magnitudes around the polar regions. Among these inwardly extracellular solute fluxes, the Na^+^ fluxes were seemed to be dominant followed closely by Cl^-^ fluxes. Conversely, the solute effluxes appear to be via the intracellular space and seemed to be more pronounced around the equatorial region of the lens. The K^+^ fluxes were found to be the primary intracellular fluxes, caused mainly by exterior Na^+^/K^+^ ATPase pumps. The net effect of these influx and effluxes were thought to be best explained by the calculated net current densities. The pattern of these net current densities at the surface of the lens was similar to previous experimental findings [5,6,52]. Same fields modelled inside the lens found to be in agreement with the microcirculation theory of the lens [3,9,16,61].

This study brings together all the available experimental and theoretical data on the fluid dynamics of the ocular lens in order to create a comprehensive 3D model of this tissue. Previous studies have investigated the links between the steady state fluxes in the lens and its physiological homeostasis [62-65]. Using our computational model, we would be able to study the connections between the biodynamic natures of these perturbations and their functional consequences.

## Competing interests

The authors declare that they have no competing interests.

## Authors' contributions

**EV** created the computational model and drafted this manuscript. **DM** checked the integrity of the equations and numerical presentations. **MJ** checked the numerical outcomes of the model and edited the paper. **PD** conceived the manuscript; final edited it and approved the final version. All authors read and approved the final manuscript.
